# Establishing Tunable Genetic Logic Gates with Versatile Dynamic Performance by Varying Regulatory Parameters

**DOI:** 10.1021/acssynbio.3c00554

**Published:** 2023-11-30

**Authors:** Tian Jiang, Yuxi Teng, Chenyi Li, Qi Gan, Jianli Zhang, Yusong Zou, Bhaven Kalpesh Desai, Yajun Yan

**Affiliations:** † School of Chemical, Materials, and Biomedical Engineering, College of Engineering, The University of Georgia, Athens, Georgia 30602, United States

**Keywords:** logic gate, BUF, AND, NOT, biosensor, *p*-coumaric acid

## Abstract

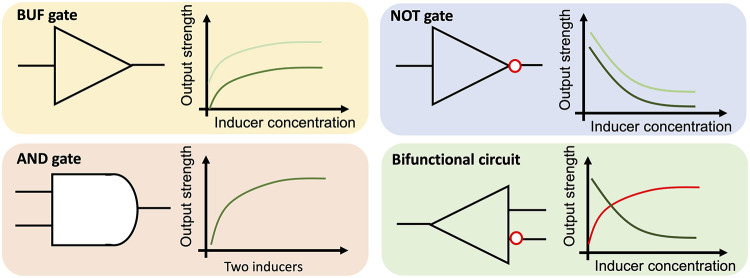

Genetic logic gates can be employed in metabolic engineering and synthetic biology to regulate gene expression based on diverse inputs. Design of tunable genetic logic gates with versatile dynamic performance is essential for expanding the usability of these toolsets. Here, using the *p*-coumaric acid biosensor system as a proof-of-concept, we initially investigated the parameters influencing the buffer (BUF) genetic logic gates. Subsequently, integrating binding sequences from the *p*-coumaric acid biosensor system and tetR or lacI regulation systems into a constitutive promoter yielded AND genetic logic gates. Additionally, characterized antisense RNAs (asRNAs) or single guide RNAs (sgRNAs) with various repression efficiencies were combined with BUF gates to construct a suite of *p*-coumaric acid-triggered NOT genetic logic gates. Finally, the designed BUF and NOT gates were combined to construct bifunctional genetic circuits that were subjected to orthogonality evaluation. The genetic logic gates established in this study can serve as valuable tools in future applications of metabolic engineering and synthetic biology.

## 1 Introduction

Synthetic biology involves the characterization of biobricks, such as promoter, RBS, and enzyme, to create new biological systems and redesign existing systems that can perform specific functions.^1^ The core of synthetic biology is to construct tunable and programmable genetic systems by the assembly of standard gene devices, such as genetic logic gates, oscillators, toggle switches, and feedback loops.^2^ Previous studies have successfully demonstrated the construction of a bistable switch and self-sustaining oscillations, which represent the first attempt to construct tunable genetic circuits in *E. coli*.^3,4^ These gene devices provide bases for the development of complex genetic systems that can be used for programmable gene regulation, leading to novel applications in metabolic engineering, such as the dynamic and autonomous regulation of metabolic networks to produce valuable products.^5^

Standard and modular genetic logic gates, regulating pathway performance based on the input signals, are crucial for constructing robust genetic systems in metabolic engineering and synthetic biology.^6^ Taking inspiration from the behavior of electrical devices, genetic logic gates can be designed by linking various transcriptional factor-based biosensors to achieve customized cellular behavior in a logical manner.^7^ The inducers can function as input signals to trigger target logic gates with regulator proteins, and target protein concentration can serve as the output.^8^ Several kinds of genetic logic gates have been designed and characterized, such as AND gate,^9,10^ NOT gate, and NAND gate.^11^

While electronic technology has reached a high level of standardization, genetic logic gates are still in the early stages of development, with only a limited number of standardized logic gates currently available. Additionally, the construction of an AND genetic logic gate typically involves splitting the regulators into two components, each controlled by distinct inducible promoters. Activation of the output promoter for downstream gene expression occurs only when both inducers are present, necessitating the development of a specific regulation system involving two regulators or the design of regulators that can be split into two independent components. Moreover, the lack of orthogonality among genetic logic gates has posed a challenge for assembling complex logic systems that can regulate sophisticated biological pathways. Researchers are actively working to expand the library of standard genetic logic gates and to develop new methods for creating more versatile and orthogonal logic gates.^12^ In this paper, using the *p*-coumaric acid biosensor system as a proof-of-concept, we first explored the parameters which affect the performance of BUF gates by controlling the expression levels of regulator K127Y and reporter eGFP. Second, AND genetic logic gates were designed by combining binding sequences of two regulators in one promoter. Additionally, characterized asRNAs or sgRNAs were combined with the BUF gates to develop *p*-coumaric acid-triggered NOT genetic logic gates. Furthermore, diverse BUF and NOT gates were combined to explore the orthogonality. These works provided some inspiration for the development of genetic logic gates, increasing their availability in *p*-coumaric acid derived pathways to autonomously coordinate gene activation and repression.

## 2 Methods and Materials

### 2.1 Strains and Plasmids

High copy plasmids pHA-MCS, pHA-eGFP-MCS, and pZE-eGFP, and medium copy plasmids pMK-MCS, pMK-eGFP-MCS, and pCS-eGFP were used for plasmids construction. *E. coli* XL1-Blue was used for plasmid construction. *E. coli* BW25113 (F′) and *E. coli* BW25113 (F′)::dCas9 were used for genetic logic gates characterization. Plasmids and strains used in this paper are shown in Table 1.

**Table 1:** List of Plasmids and Strains Used in This Study

strains	genotype	reference
*E. coli* XL1-Blue	*recA1 endA1gyrA96thi-1hsdR17supE44relA1lac[F′ proAB lacIqZDM15Tn10 (TetR)]*	Stratagene
*E. coli* BW25113 (F′)	*rrnBT14 ΔlacZWJ16 hsdR514 ΔaraBADAH33 ΔrhaBADLD78 F′ [traD36 proAB lacIqZΔM15 Tn10(Tetr)]*	(13)
*E. coli* BW25113(F′)::dCas9	*E. coli* BW25113(F′) with the pLlacO1-controlled dCas9 from *Streptococcus pyogenes* integrated at the low-expression *dkgB* locus	(14)

plasmids	description	reference
pMK-eGFP-MCS	pLlacO1; eGFP; multiple cloning site; *p15A ori*; *Kan*^*R*^	this study
pMK-MCS	pLlacO1; multiple cloning site; *p15A ori*; *Kan*^*R*^	this study
pCS-eGFP	pLlacO1; eGFP; *p15A ori*; *Kan*^*R*^	(15)
pCS27	pLlacO1; *p15A ori*; *Kan*^*R*^	(16)
pHA-eGFP-MCS	pLlacO1; eGFP; multiple cloning site; *ColE1 ori*; *Amp*^*R*^	(17)
pHA-MCS	pLlacO1; multiple cloning site; *ColE1 ori*; *Amp*^*R*^	(18)
pZE-eGFP	pLlacO1; eGFP; *ColE1 ori*; *Amp*^*R*^	(19)
pZE12-*luc*	pLlacO-1; luc; *ColE1 ori*; *Amp*^*R*^	(20)
pZE-PT	pZE12-luc harboring PT template	(21)
pZE-P9-RBS0.3- eGFP	pZE12-*luc* carrying promoter P9, RBS0.3, and *egfp* gene	this study
pZE-P9-RBS0.6-eGFP	pZE12-*luc* carrying promoter P9, RBS0.6, and *egfp* gene	this study
pZE-P9-RBS0.9-eGFP	pZE12-*luc* carrying promoter P9, RBS0.9, and *egfp* gene	this study
pZE-P9-RBS1.0-eGFP	pZE12-*luc* carrying promoter P9, RBS1.0, and *egfp* gene	(18)
pZE-P9-RBS1.2-eGFP	pZE12-*luc* carrying promoter P9, RBS1.2, and *egfp* gene	this study
pCS-lpp0.2-RBS0.3-K127Y	pCS27 carrying the promoter lpp0.2, RBS0.3, and regulator K127Y	this study
pCS-lpp0.2-RBS0.6-K127Y	pCS27 carrying the promoter lpp0.2, RBS0.6, and regulator K127Y	this study
pCS-lpp0.2-RBS0.9-K127Y	pCS27 carrying the promoter lpp0.2, RBS0.9, and regulator K127Y	this study
pCS-lpp0.2-RBS1.0-K127Y	pCS27 carrying the promoter lpp0.2, RBS1.0, and regulator K127Y	(18)
pCS-lpp0.2-RBS1.2-K127Y	pCS27 carrying the promoter lpp0.2, RBS1.2, and regulator K127Y	this study
pHA-version 1-eGFP	pHA-MCS carrying the AND promoter version 1 and *egfp* gene	this study
pHA-version 2-eGFP	pHA-MCS carrying the AND promoter version 2 and *egfp* gene	this study
pHA-version 3-eGFP	pHA-MCS carrying the AND promoter version 3 and *egfp* gene	this study
pHA-version 4-eGFP	pHA-MCS carrying the AND promoter version 4 and *egfp* gene	this study
pHA-version 5-eGFP	pHA-MCS carrying the AND promoter version 5 and *egfp* gene	this study
pZE-P_fdeA_-eGFP	pZE12-*luc* carrying promoter P_fdeA_ and *egfp* gene	(18)
pCS-lpp1.0-FdeR	pCS27 carrying promoter lpp1.0 and regulator FdeR	(18)
pCS-lpp0.2-K127Y-lpp1.0-FdeR	pCS-lpp0.2-K127Y carrying the operon with promoter lpp1.0, regulator FdeR, and terminator T1	this study
pZE-P9-tetR-eGFP	pZE12-*luc* carrying AND promoter P9-tetR and *egfp* gene	this study
pZE-P9-lacI-eGFP	pZE12-*luc* carrying AND promoter P9-lacI and *egfp* gene	this study
pCS-lpp0.2-tetR	pCS27 carrying promoter lpp0.2 and regulator tetR	this study
pCS-lpp0.2-K127Y-lpp0.2-tetR	pCS-lpp0.2-K127Y carrying the operon with promoter lpp0.2, regulator tetR, and terminator T1	this study
pZE-P9-asegfp20	pZE12-*luc* carrying promoter P9 and 20 bp DNA transcribed to RNA targeting *egfp*	this study
pZE-P9-asegfp100	pZE12-*luc* carrying promoter P9 and 100 bp DNA transcribed to RNA targeting *egfp*	this study
pMK-P9-sgRNA10	pMK-MCS carrying promoter P9 and sgRNA10	this study
pMK-P9-sgRNA12	pMK-MCS carrying promoter P9 and sgRNA12	this study
pMK-eGFP-MCS-lpp0.2-K127Y	pMK-eGFP-MCS carrying the operon with promoter lpp0.2, regulator K127Y, and terminator T1	this study
pMK-P9-sgRNA10-lpp0.2-K127Y	pMK-P9-sgRNA10 carrying the operon with promoter lpp0.2, regulator K127Y, and terminator T1	this study
pMK-P9-sgRNA12-lpp0.2-K127Y	pMK-P9-sgRNA12 carrying the operon with promoter lpp0.2, regulator K127Y, and terminator T1	this study
pZE-P9-RBS0.3-RFP	pZE12-*luc* carrying promoter P9, RBS0.3, and *rfp* gene	this study
pZE-P9-RBS0.6-RFP	pZE12-*luc* carrying promoter P9, RBS0.6, and *rfp* gene	this study
pZE-P9-RBS1.0-RFP	pZE12-*luc* carrying promoter P9, RBS1.0, and *rfp* gene	this study
pZE-P9-RBS0.3-RFP-P9-asegfp20	pZE-P9-RBS0.3-RFP carrying promoter P9 and 20 bp DNA transcribed to RNA targeting *egfp*	this study
pZE-P9-RBS0.6-RFP-P9-asegfp20	pZE-P9-RBS0.6-RFP carrying promoter P9 and 20 bp DNA transcribed to RNA targeting *egfp*	this study
pZE-P9-RBS1.0-RFP-P9-asegfp20	pZE-P9-RBS1.0-RFP carrying promoter P9 and 20 bp DNA transcribed to RNA targeting *egfp*	this study
pZE-P9-RBS0.3-RFP-P9-asegfp100	pZE-P9-RBS0.3-RFP carrying promoter P9 and 100 bp DNA transcribed to RNA targeting *egfp*	this study
pZE-P9-RBS0.6-RFP-P9-asegfp100	pZE-P9-RBS0.6-RFP carrying promoter P9 and 100 bp DNA transcribed to RNA targeting *egfp*	this study
pZE-P9-RBS1.0-RFP-P9-asegfp100	pZE-P9-RBS1.0-RFP carrying promoter P9 and 100 bp DNA transcribed to RNA targeting *egfp*	this study

### 2.2 Medium and Chemicals

Luria–Bertani (LB) medium containing 10 g/L NaCl, 5 g/L yeast extract, and 10 g/L tryptone was used for plasmid construction and genetic logic gates characterization. The antibiotics kanamycin and ampicillin were added into the LB medium, if necessary, with the final concentrations of 100 and 50 μg/mL, respectively. The inducers, *p*-coumaric acid and naringenin, were dissolved in methanol. Methanol was purchased from Fisher Chemicals. The tetracycline was dissolved in water. High-Fidelity Phusion DNA polymerase, restriction endonucleases, and Quick Ligation Kit were purchased from New England Biolabs (Beverly, MA, USA). Zyppy Plasmid Miniprep Kit, Zymoclean Gel DNA Recovery Kit, and DNA Clean & Concentrator-5 were purchased from Zymo Research (Irvine, CA, USA).

### 2.3 DNA Manipulation

The medium copy plasmid pMK-MCS was constructed in our lab, which contains a p15A origin, a kanamycin resistance gene, pLlacO1 promoter, and T1 terminator as reported in the previous study.^20^ The plasmid also carries a synthetic multicloning site (MCS) that sequentially contains the recognition sites of Acc65I, NdeI, BsrGI, *Sal*I, ClaI, *Hin*dIII, NheI, *Bam*HI, and MluI. pMK-eGFP-MCS was constructed by inserting the coding sequence of eGFP into pMK-MCS using Acc65I and *Sal*I. The constructive promoters lpp0.2 and lpp1.0 were used in this paper to control related genes expression.^22^ To characterize RBS mutants, the plasmids pZE-P9-RBS (0.3, 0.6, 0.9, 1.2)-eGFP were constructed by inserting the DNA fragment RBS (0.3, 0.6, 0.9, 1.2)-eGFP into pZE-P9-RBS1.0-eGFP, respectively, using *Kpn*I and XbaI. To construct the plasmids pCS-lpp0.2-RBS (0.3, 0.6, 0.9, 1.2)-K127Y, the gene fragments RBS (0.3, 0.6, 0.9, 1.2)-K127Y were inserted into plasmid pCS-lpp0.2-RBS1.0-K127Y using *Kpn*I and *Bam*HI. For the construction of five versions of AND genetic gates, the gene fragments version1–5-eGFP, which consist of different AND promoters followed by the *egfp* gene, respectively, were inserted into the vector pHA-MCS, respectively, using the XhoI and XbaI. For the construction of pZE-P9-tetR-eGFP and pZE-P9-lacI-eGFP, the gene fragment P9-tetR-eGFP and P9-lacI-eGFP, which containing P9 promoter, tetR or lacI regulator, and *egfp* gene, were inserted into the vector pHA-MCS, respectively, using the XhoI and XbaI. The plasmid pCS-lpp0.2-tetR was constructed in one of our unpublished papers. To construct the plasmid pCS-lpp0.2-K127Y-lpp0.2-tetR, the operon lpp0.2-tetR-terminator, containing promoter lpp0.2, tetR regulator, and terminator, was inserted into the plasmid pCS-lpp0.2-K127Y using the BspHI and XhoI. The plasmid pCS-lpp0.2-K127Y-lpp1.0-FdeR was constructed by placing the lpp1.0-FdeR-terminator operon, containing promoter lpp1.0, regulator FdeR, and terminator, in pCS-lpp0.2-K127Y using AatII and XhoI. To construct the plasmid pZE-P9-asegfp20, the gene fragment P9-asegfp20, containing the P9 promoter, a 35-bp paired termini (PT) sequence, and a 20-bp DNA sequence transcribed to RNA targeting the mRNA of *egfp*, was placed in the vector pZE12-PT using XhoI and *Bam*HI. The plasmid pZE-PT was constructed in our lab previously.^21^ To construct the plasmid pZE-P9-asegfp100, the gene fragment asegfp100, a 100-bp DNA sequence transcribed to RNA targeting the mRNA of *egfp*, was inserted into the plasmid pZE-P9-asfabD using *Kpn*I and *Bam*HI, and the pZE-P9-asfabD was constructed in our previous research.^18^ To construct the plasmids pMK-P9-sgRNA10 and pMK-P9-sgRNA12, the gene fragments P9-sgRNA10 and P9-sgRNA12 with terminator and cas9 handle sequence were inserted into the vector pMK-MCS using BspHI and *Bam*HI. To construct the plasmid pMK-eGFP-MCS-lpp0.2-K127Y, the operon lpp0.2-K127Y-terminator was placed in pMK-eGFP-MCS using BspHI and XhoI. The plasmids pMK-P9-sgRNA10-lpp0.2-K127Y and pMK-P9-sgRNA12-lpp0.2-K127Y were constructed by placing the operon lpp0.2-K127Y-terminator, containing promoter lpp0.2, regulator K127Y, and terminator, in pMK-P9-sgRNA10 and pMK-P9-sgRNA12, respectively, using BspHI and XhoI. The plasmids pZE-P9-RBS0.3/0.6/1.0-RFP were constructed by placing the *rfp* gene fragment in pZE-P9-RBS0.3/0.6/1.0-eGFP, respectively, using *Kpn*I and XbaI. The six plasmids pZE-P9-RBS0.3/0.6/1.0-RFP-P9-asegfp20/100 were constructed by placing the operon P9-asegfp20/100 into pZE-P9-RBS0.3/0.6/1.0-RFP, respectively, using SpeI and SacI.

### 2.4 Genetic Logic Gates Characterization

To characterize the genetic logic gates, the related plasmids were transformed into *E. coli* BW25113 (F′) or *E. coli* BW25113 (F′)::dCas9. We randomly selected three independent transformants and cultivate them in 3.5 mL of LB medium with appropriate antibiotics. The seeds were incubated in a New Brunswick Excella E24 shaker at 37 °C with a shaking speed of 270 rpm for approximately 12 h. Subsequently, we transferred 150 μL of the seeds into 3.5 mL of LB medium. Once the OD_600_ reaches around 0.4, which usually takes roughly 1.5 h of cultivation, different concentrations of inducers were added to the medium. After 24 h of cultivation, the cultures were sampled to measure the fluorescence intensity and cell density.

### 2.5 Fluorescence Intensity Assay

To measure the fluorescence intensity, we used the Synergy HT plate reader from Biotek. First, the samples were diluted by 4 times using 150 μL of DI water and 50 μL of the sample and then transferring the mixture to a 96-well plate (Corning 96-well Flat Clear Bottom Black Polystyrene TC-treated Microplates, Corning 3603). The green fluorescence intensity was detected by using an excitation filter of 485/20 nm and an emission filter of 528/20 nm. The red fluorescence intensity was detected by using an excitation filter of 530/25 nm and an emission filter of 590/35 nm. To calculate the unit eGFP or RFP expression levels (RFU/OD_600_), the fluorescence intensities were normalized with the corresponding cell densities using the following formula.
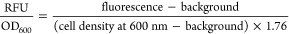


## 3 Results

### 3.1 Investigation of Parameters That Affect the Performance of BUF Gate

**Figure 1:**
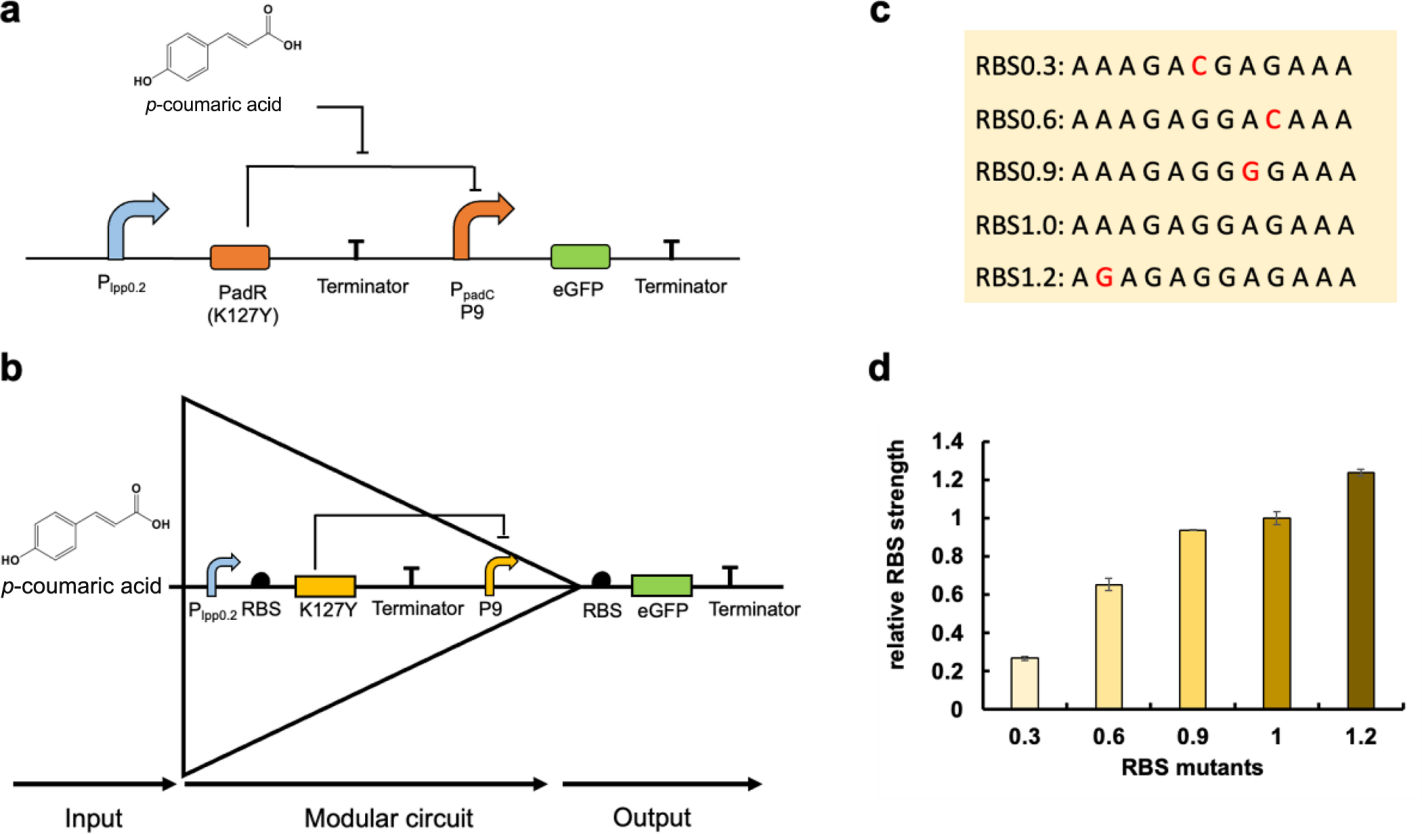
Design of BUF gates. (a) The mechanism of the *p*-coumaric acid biosensor system; promoter P9 is repressed by regulator K127Y to inhibit the reporter gene *egfp*. The addition of *p*-coumaric acid can combine with K127Y to release the repression toward P9 that causes eGFP expression. (b) The mechanism of the *p*-coumaric acid-triggered BUF gate; *p*-coumaric acid is considered as the input and eGFP is the output. The existence of *p*-coumaric acid can open the BUF gate for eGFP expression. On the contrary, the BUF gate cannot be turned on without *p*-coumaric acid addition. (c) The detailed sequence of the RBS wild type (RBS1.0) and its mutants. (d) The relative RBS strength. All data are reported as mean ± s.d. from three biologically independent experiments (*n* = 3). Error bars are defined as s.d.

In the *p*-coumaric acid biosensor system, PadR serves as a transcriptional repressor, inhibiting the expression of phenolic acid decarboxylase (PadC) in *Bacillus subtilis*. This inhibition occurs through binding of PadR to the specific sequence in P_padC_, which is the promoter of the *padC* gene, effectively blocking the access of RNA polymerase. In the presence of *p*-coumaric acid, the repressor undergoes a conformational change upon binding to these compounds, releasing the inhibition.^23,24^ Our previous research optimized and engineered this biosensor system,^15,18^ which led to the development of the PadR variant (K127Y) demonstrating enhanced sensitivity, and the P_padC_ variant (P9) exhibiting increased strength (Figure 1a). With the well-characterized and optimized PadR-P_padC_ (K127Y–P9) sensor system, it becomes feasible to achieve *p*-coumaric acid-triggered genetic logic gates by combining various genetic elements to achieve diverse output.

Within the context of a genetic logic gate, the *p*-coumaric acid biosensor system functions as a buffer (BUF) gate with *p*-coumaric acid as the input and eGFP expression level as the output. Upon the addition of *p*-coumaric acid, the BUF gate is triggered to open, allowing the expression of P9-controlled eGFP through releasing the repression from K127Y (Figure 1b). Here, we aim to investigate how different parameters affect the dynamic outputs of the BUF gates and construct different versions of BUF gates with diverse dynamic performance. To achieve this, we characterized different RBS sequences from the iGEM library (http://parts.igem.org/Part:BBa_K1676100), as shown in Figure 1c. By employing eGFP as the reporter, we characterized these RBS variants with gradually decreased strength, which can be used in the following genetic logic gates (Figure 1d).

**Figure 2:**
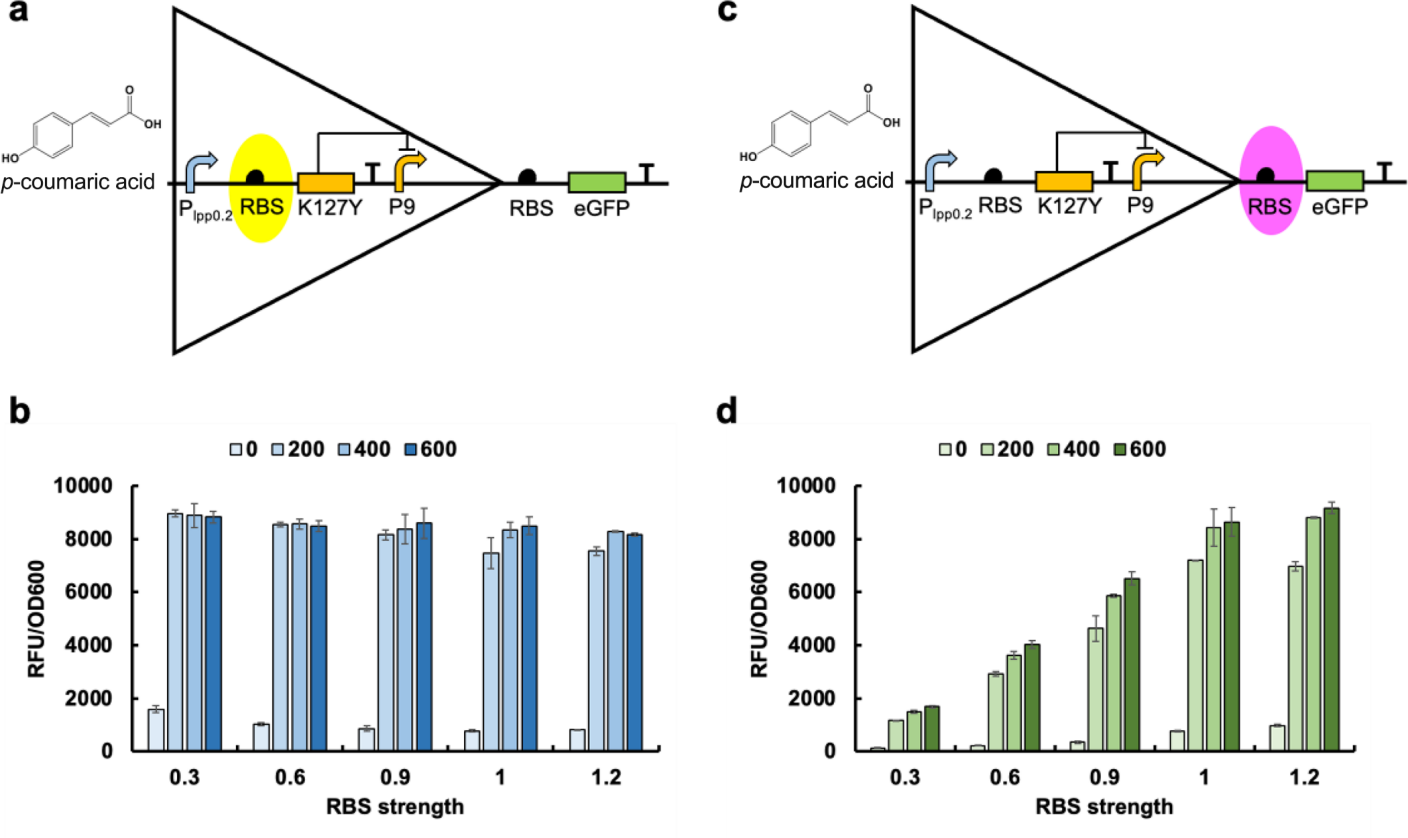
Dynamic performance of the BUF gates. (a) Design of BUF gates with varying regulator expression level. (b) The dynamic performance of BUF gates with changed regulator expression. (c) The design of BUF gates with varying output gene expression. (d) The dynamic performance of BUF gates with changed output gene expression. 0:0 mg/L *p*-coumaric acid; 200:200 mg/L *p*-coumaric acid; 400:400 mg/L *p*-coumaric acid; 600:600 mg/L *p*-coumaric acid. All data are reported as mean ± s.d. from three biologically independent experiments (*n* = 3). Error bars are defined as s.d.

Diverse BUF gates were designed by placing characterized RBS mutants upstream of regulator K127Y (Figure 2a). We hypothesized that the dynamic performance of these BUF gates would exhibit gradual changes in response to variations in the expression level of the regulator upon the addition of *p*-coumaric acid. However, the results depicted in Figure 2b revealed that regardless of the RBS mutants used, the dynamic behavior of the BUF gates exhibited similar trends upon exposure to different concentrations of *p*-coumaric acid. The potential reason is that the P9 promoter might already be saturated and inhibited at a low expression level (RBS 0.3) of K127Y. Consequently, increasing the expression level further does not lead to improved inhibitory efficiency, nor does the addition of *p*-coumaric acid result in higher activation strength. Instead, altering the strength of the RBS within a specific range to prevent P9 from reaching saturation could potentially induce distinct dynamic performances. However, it is worth noting that low RBS strength, combined with unsaturated inhibition of P9, might also contribute to system leakage. Especially, we observed a 50% higher leakage in the BUF gate group with a RBS of 0.3 compared to the group with a RBS of 0.6 (Figure 2b). These findings underscore the intricate interplay between regulator expression levels and promoter saturation, both of which significantly impact the dynamic behavior of the BUF gates.

Next, we employed RBS mutants before the reporter gene *egfp* to design another group of BUF gates (Figure 2c), based on the hypothesis that changed eGFP expression would lead to gradual changes in dynamic output with the addition of *p*-coumaric acid. As depicted in Figure 2d, these BUF gates can be effectively suppressed in the absence of *p*-coumaric acid. With an increasing RBS strength, the leakage of the BUF gates also escalates. Upon introducing varying concentrations of *p*-coumaric acid, all BUF gates exhibit different degrees of opening, with the output intensity gradually increasing alongside RBS strength. The addition of 600 mg/L *p*-coumaric acid resulted in a significant increase in output strength, with various fold changes of 12, 17, 18, 11, and 9 observed in the five BUF gates that feature progressively stronger RBSs before the eGFP gene. These results demonstrate that fine-tuning the expression level of the reporter gene enables precise control of the BUF gate performance, resulting in distinct phenotypes that can be utilized in various regulatory systems. The BUF gates we developed can autonomously coordinate gene activation based on *p*-coumaric acid concentrations in related pathways in future studies. After investigating the impact of fine-tuning the regulator and output gene expression, we can conclude that regulating the output gene is an effective strategy for designing versatile BUF gates with adjustable dynamic performance. However, achieving precise regulator-based control is challenging given the intricate interplay between regulator expression level and promoter saturation.

### 3.2 The Design and Characterization of Hybrid Promoter-Based AND Genetic Logic Gates

**Figure 3:**
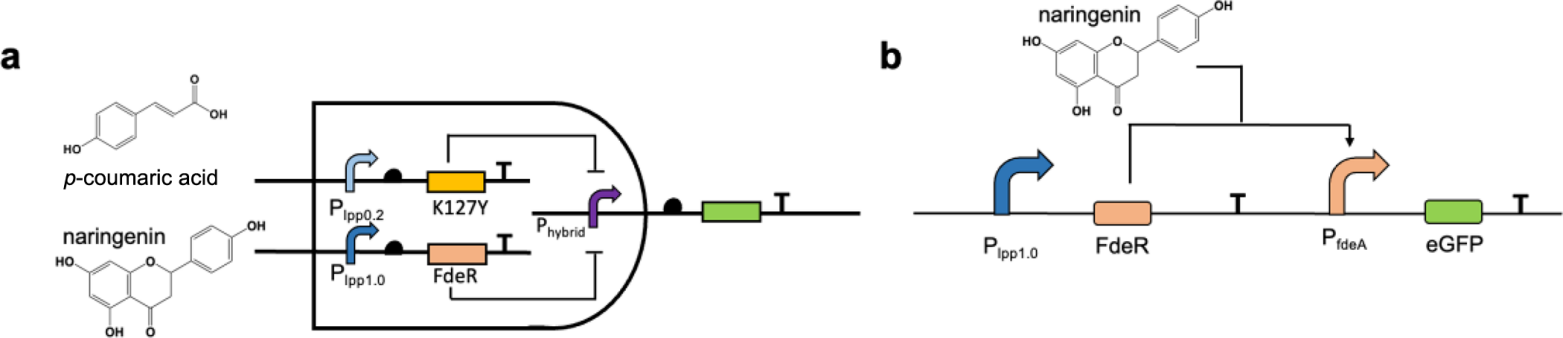
Mechanism of the genetic AND logic gate. (a) By placing the binding sequence of K127Y and FdeR in one promoter, the resulting hybrid promoter can be repressed by K127Y and FdeR, and only the addition of *p*-coumaric acid and naringenin simultaneously can activate the expression of a downstream gene. (b) The mechanism of naringenin biosensor system. Without naringenin, the eGFP expression will be repressed. The added naringenin combines with FdeR to initiate the expression of eGFP.

The AND gate generates a high output only when all of its inputs are in a high state. In *E. coli*, this gate is usually constructed by dividing the regulators into two components that are controlled by different inducible promoters. Only when both inducers are present can the two components come together to activate the output promoter for downstream gene expression.^11^ The success of this type of AND genetic logic gate relies on the development of a specific regulation system that requires two regulators or the design of a regulator that can be split into two independent components. Here, we proposed an alternative approach that focused on the engineering of the output promoter. Lutz et al. designed an inducible promoter P_lac/ara-1_ by integrating the binding sequence of arabinose and IPTG induction systems into pL promoter.^20^ The promoter can be repressed when the transcriptional activator araC and transcriptional repressor lacI combine to form the related positions in P_lac/ara-1_. Promoter transcription could only commence upon the addition of both arabinose and IPTG. However, their results showed that adding IPTG alone led to significant leakage in promoter activity, with a 125-fold increase, and the effect of adding arabinose individually was not explored. Inspired by previous studies, we hypothesized that, by combining the binding sequences of two independent repressors into one promoter, the resulting hybrid promoter will be repressed simultaneously. Only the presence of both inducers can activate the promoter and initiate gene expression (Figure 3a). This strategy with the hybrid promoter simplifies the elements in AND genetic logic gates and offers a new perspective for AND gate design in synthetic biology.

**Figure 4:**
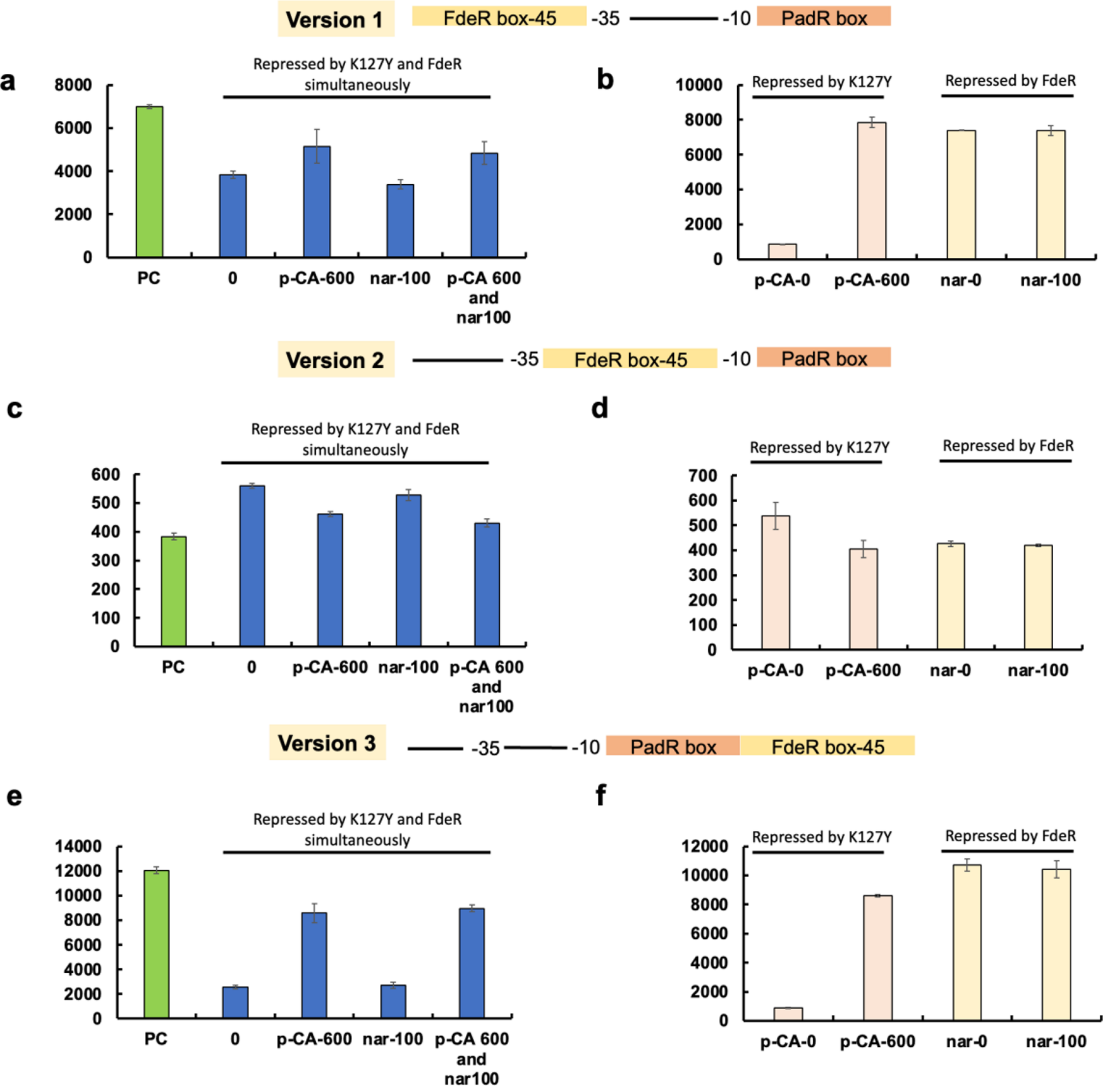
Design and characterization of K127Y and FdeR-based AND genetic logic gates with a short FdeR binding sequence. **Version 1**: the 45 bp FdeR binding box was placed before the −35 region and the K127Y binding box was positioned behind the −10 region to replace related DNA sequences in the pL promoter. **Version 2**: the 45 bp FdeR binding box was placed between −35 and −10 regions, and the K127Y binding box was positioned behind the −10 region to replace related DNA sequences in the pL promoter. **Version 3**: the K127Y binding box was positioned behind the −10 region and the 45 bp FdeR binding box was placed behind the K127Y binding box to replace related DNA sequences in the pL promoter. (a) The version 1 promoter was repressed by K127Y and FdeR simultaneously. Naringenin and *p*-coumaric acid were added individually or simultaneously to activate the promoter. (b) Repressed by K127Y: The version 1 promoter was repressed by K127Y and *p*-coumaric acid was added as inducer. Repressed by FdeR: The version 1 promoter was repressed by FdeR and naringenin was added as inducer. (c) The version 2 promoter was repressed by K127Y and FdeR simultaneously. Naringenin and *p*-coumaric acid were added individually or simultaneously to release the promoter. (d) Repressed by K127Y: The version 2 promoter was repressed by K127Y and *p*-coumaric acid was added as inducer. Repressed by FdeR: The version 2 promoter was repressed by FdeR and naringenin was added as inducer. (e) The version 3 promoter was repressed by K127Y and FdeR simultaneously. Naringenin and *p*-coumaric acid were added individually or simultaneously to release the promoter. (f) Repressed by K127Y: The version 3 promoter was repressed by K127Y and *p*-coumaric acid was added as inducer. Repressed by FdeR: The version 3 promoter was repressed by FdeR and naringenin was added as inducer. PC: no repression on the promoter; 0: no inducer was added; *p*-CA-600:600 mg/L *p*-coumaric acid; nar-100:100 mg/L naringenin; *p*-CA-600 and nar-100:600 mg/L *p*-coumaric acid and 100 mg/L naringenin. All data are reported as mean ± s.d. from three biologically independent experiments (*n* = 3). Error bars are defined as s.d.

In our previous research, the FdeR-P_fdeA_ biosensor system was characterized which can respond to different concentrations of naringenin^18^ (Figure 3b). The binding box of FdeR has been preliminarily characterized with the repeat sequence of T-N11-A.^25,26^ Building on this knowledge and using the *p*-coumaric acid and naringenin biosensor system as a proof-of-concept, we combined the binding sequences of K127Y and FdeR into one promoter to explore the possibility of constructing AND genetic logic gates. Here, we designed three hybrid promoters by replacing the related DNA sequence in the constitutive promoter pL with the FdeR and K127Y binding boxes, as shown in Figure 4. The promoter version 2 lost most of its activity and, notably, it cannot be repressed by K127Y and FdeR simultaneously or independently (Figure 4c,d). The potential reason is that the distance between the −35 and −10 regions is crucial for promoter activity, and the long sequence of the FdeR binding box (45 bp) disrupted the promoter function. In the absence of regulators, both promoter version 1 and version 3 functioned successfully (Figure 4a,e). However, 46% and 80% of the promoter activity was repressed in the presence of K127Y and FdeR in version 1 and version 3, respectively. The addition of *p*-coumaric acid alone was sufficient to fully release the repression on the two promoters. The coaddition of *p*-coumaric acid and naringenin did not lead to increased activation. These results suggested that FdeR may not play a role in repression. To further support the speculation, K127Y and FdeR were transformed with promoter versions 1 and 3 within one cell, respectively. As expected, in the group cotransformed with K127Y, the promoters were repressed by K127Y and activated by *p*-coumaric acid. However, no repression was achieved in the presence of FdeR (Figure 3b,f). These results provide strong evidence that K127Y is the repressor for promoter version 1 and version 3 repression, and FdeR does not contribute to repression in this context.

**Figure 5:**
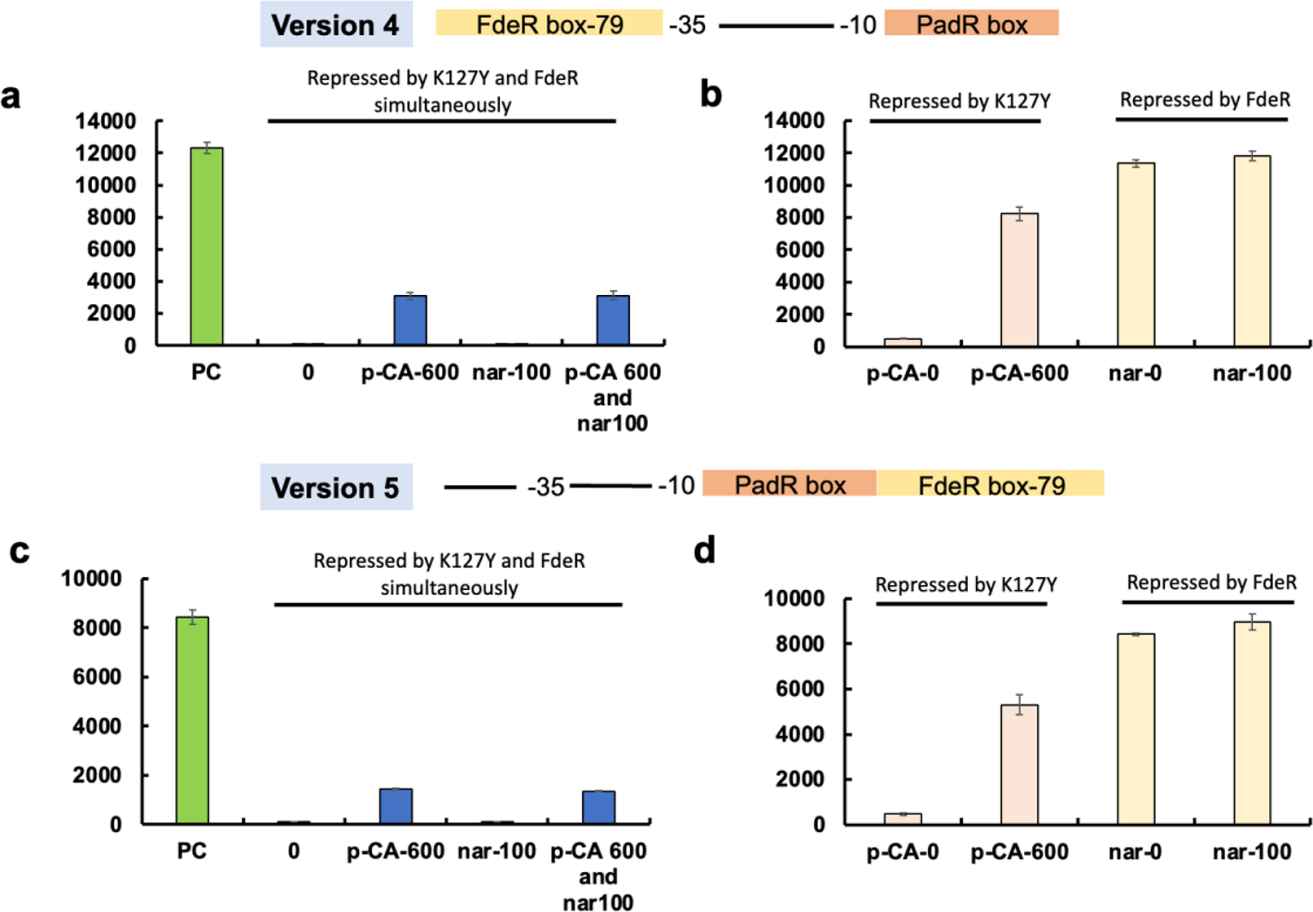
Design and characterization of K127Y and FdeR-based AND genetic logic gates with a long FdeR binding box (79 bp). **Version 4**: the 79 bp FdeR binding box was placed before the −35 region, and the K127Y binding box was positioned behind the −10 region to replace related DNA sequences in the pL promoter. **Version 5**: the K127Y binding box was positioned behind the −10 region and the 79 bp FdeR binding box was placed behind the K127Y binding box to replace related DNA sequence in the pL promoter. (a) The version 4 promoter was repressed by K127Y and FdeR simultaneously. Naringenin and *p*-coumaric acid were added individually or simultaneously to release the promoter. (b) Repressed by K127Y: The version 4 promoter was repressed by K127Y and *p*-coumaric acid was added as inducer. Repressed by FdeR: The version 4 promoter was repressed by FdeR and naringenin was added as inducer. (c) The version 5 promoter was repressed by K127Y and FdeR simultaneously. Naringenin and *p*-coumaric acid were added individually or simultaneously to release the promoter. (d) Repressed by K127Y: The version 5 promoter was repressed by K127Y and *p*-coumaric acid was added as inducer. Repressed by FdeR: The version 5 promoter was repressed by FdeR and naringenin was added as inducer. PC: no repression on the promoter; 0: no inducer was added; *p*-CA-600: 600 mg/L *p*-coumaric acid; nar-100: 100 mg/L naringenin; *p*-CA-600 and nar-100: 600 mg/L *p*-coumaric acid and 100 mg/L naringenin. All data are reported as mean ± s.d. from three biologically independent experiments (*n* = 3). Error bars are defined as s.d. All data are reported as mean ± s.d. from three biologically independent experiments (*n* = 3). Error bars are defined as s.d.

We speculated that the 45 bp binding box initially used may not include all of the T-N11-A sequence required for effective FdeR binding. Therefore, the FdeR binding sequence was extended to 79 bp, containing 11 repeats of overlapped T-N11-A. We designed version 4 and version 5 hybrid promoters with the 79 bp binding sequence positioned before the −35 region and behind the −10 region in pL, respectively (Figure 5). However, even in the presence of *p*-coumaric acid individually, parts of the repression could still be independently released. The coaddition of *p*-coumaric acid and naringenin did not lead to further activation (Figure 5a,c). Additionally, the promoter version 4 and version 5 can be repressed when K127Y was individually transformed and activated by *p*-coumaric acid, but individual transformation of FdeR did not show any repression toward the two promoters (Figure 5b,d). In summary, our attempts to construct AND genetic logic gates using the combination of regulator binding boxes from *p*-coumaric acid and naringenin biosensor systems were not successful. The potential reason is that the FdeR binding box we utilized cannot function in a plug-and-play manner, limiting the efficiency of FdeR repression.

**Figure 6:**
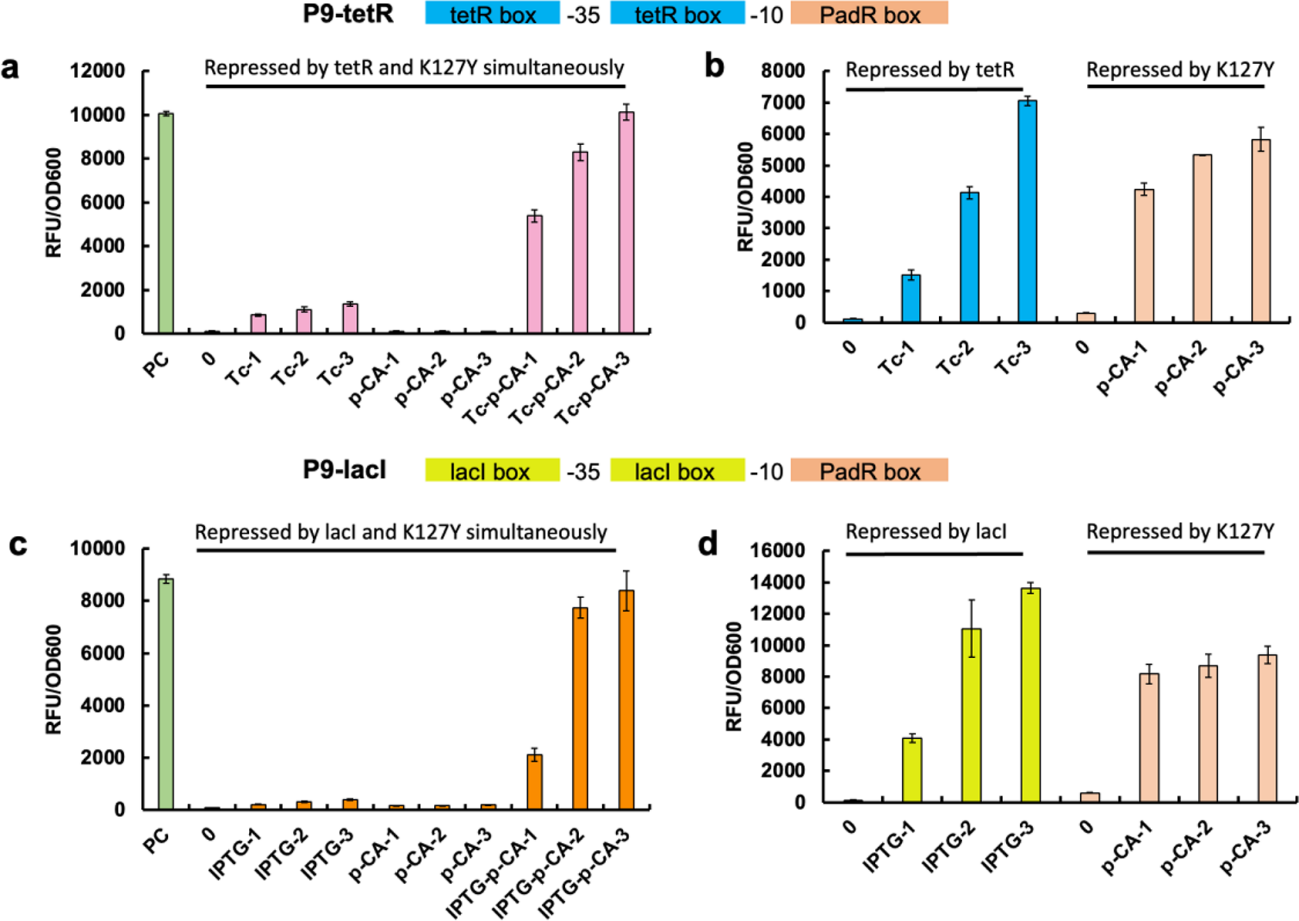
Design and characterization of K127Y and tetR or lacI triggered AND promoters. **P9-tetR**: K127Y binding box was placed behind the −10 region, and the tetR binding box was placed before the −35 region and between the −35 and −10 regions to replace the related sequence in the pL promoter. **P9-lacI**: K127Y binding box was placed behind the −10 region, and the lacI binding box was placed before the −35 region and between the −35 and −10 regions to replace the related sequence in the pL promoter. (a) The promoter P9-tetR was simultaneously repressed by K127Y and tetR. Naringenin and tetracycline were added exclusively or simultaneously to release the promoter repression. (b) Repressed by tetR: the hybrid promoter P9-tetR was repressed by tetR and tetracycline was added as inducer; Repressed by K127Y: the hybrid promoter P9-tetR was repressed by K127Y and *p*-coumaric acid was added as inducer. (c) The promoter P9-tetR was repressed by K127Y and lacI simultaneously. Naringenin and IPTG were added exclusively or simultaneously to release the promoter repression. (d) Repressed by lacI: the hybrid promoter P9-lacI was repressed by lacI and IPTG was added as inducer; Repressed by K127Y: the hybrid promoter P9-lacI was repressed by K127Y and *p*-coumaric acid was added as inducer. PC: no repression on the promoter; 0: no inducer was added; Tc-1: 2 mg/L tetracycline; Tc-2: 4 mg/L tetracycline; Tc-3: 8 mg/L tetracycline; *p*-CA-1: 200 mg/L *p*-coumaric acid; *p*-CA-2: 400 mg/L *p*-coumaric acid; *p*-CA-3: 600 mg/L *p*-coumaric acid; Tc-*p*-CA-1: 2 mg/L tetracycline and 200 mg/L *p*-coumaric acid; Tc-*p*-CA-2: 4 mg/L tetracycline and 400 mg/L *p*-coumaric acid; Tc-*p*-CA-3: 8 mg/L tetracycline and 600 mg/L *p*-coumaric acid; IPTG-1: 0.004 mM IPTG; IPTG-2: 0.008 mM IPTG; IPTG-3: 0.016 mM IPTG; IPTG-*p*-CA-1: 0.004 mM IPTG and 200 mg/L *p*-coumaric acid; IPTG-*p*-CA-2: 0.008 mM IPTG and 400 mg/L *p*-coumaric acid; IPTG-*p*-CA-3: 0.016 mM IPTG and 600 mg/L *p*-coumaric acid. All data are reported as mean ± s.d. from three biologically independent experiments (*n* = 3). Error bars are defined as s.d.

Subsequently, we hypothesized that harnessing regulatory systems with well-defined binding sequences could facilitate the construction of AND genetic logic gates. The tetracycline regulation system tetR-tetO, for instance, has been thoroughly characterized, including the identification of the tetR binding box. In this system, tetR binds to the tetO sequence, repressing downstream gene expression. The presence of tetracycline alleviates this repression, thus promoting the downstream gene expression. For the construction of AND hybrid promoter, we integrated the binding boxes of tetR and K127Y into a constitutive promoter, pL, resulting in the formation of a hybrid promoter P9-tetR (Figure 6a,b). In the absence of regulatory elements, this hybrid promoter exhibited robust activity. The introduction of tetR and K127Y repressed its activity by up to 99%. Intriguingly, individual addition of *p*-coumaric acid failed to release this repression, yet varying concentrations of tetracycline led to partial recovery of promoter activity (within 10%), showing an 11-fold increase in the output strength upon 8 mg/L tetracycline. The leakage when adding tetracycline can be attributed to the alleviation of tetR repression, facilitating RNA polymerase binding to the −35 and −10 regions, thereby triggering downstream transcription, and consequently prompting K127Y dissociation from the binding sequence downstream of the −10 regions. Notably, simultaneous coaddition of tetracycline and *p*-coumaric acid led to the release of a substantial portion of promoter activity. Particularly, the addition of 8 mg/L tetracycline and 600 mg/L *p*-coumaric acid collectively elicited the activation of up to 99% of the promoter activity, showing an 83-fold increase in the output strength. These findings underscored that only the concurrent presence of *p*-coumaric acid and tetracycline could activate the AND promoter. The successful construction of *p*-coumaric acid and tetracycline triggered AND genetic logic gate reinforced our proposition that binding boxes capable of ready integration are fundamental for the design of AND genetic logic gates.

To further bolster this proposition, the IPTG regulation system was employed to create an alternative version of the AND promoter. Within this system, the lacI repressor binds to the characterized sequence, thereby suppressing the downstream gene expression. Upon the introduction of IPTG, this repression is lifted, allowing for gene activation. Thus, we integrated the binding boxes of K127Y and lacI into the pL promoter (Figure 6). Analogous to the earlier case, the AND promoter displayed normal functionality, yet the copresence of lacI and K127Y led to a repression of up to 99% of promoter activity. Singular administration of IPTG resulted in a minor 5% leakage in the AND promoter expression. The addition of 0.016 mM IPTG caused a 4-fold increase in the output strength. However, the simultaneous presence of IPTG and *p*-coumaric acid effectively lifted the repression enforced by both lacI and K127Y. Specifically, the addition of 0.016 mM IPTG and 600 mg/L *p*-coumaric acid released as much as 95% of the promoter repression, showing a 92-fold increase in the output strength (Figure 6c). These results further corroborated our hypothesis, emphasizing the pivotal role of ready-to-use binding boxes in the construction of AND genetic logic gates. In further support of the efficacy of the constructed AND-based promoters, individual regulators tetR, K127Y, and lacI were introduced into the AND promoters. As illustrated in Figure 6b,d, these AND promoters can be repressed and activated by their respective regulators and inducers. This comprehensive array of results underscores the successful construction and operation of the AND genetic logic gates.

### 3.3 The Design and Characterization of Small RNA-Based NOT Genetic Logic Gates

**Figure 7:**
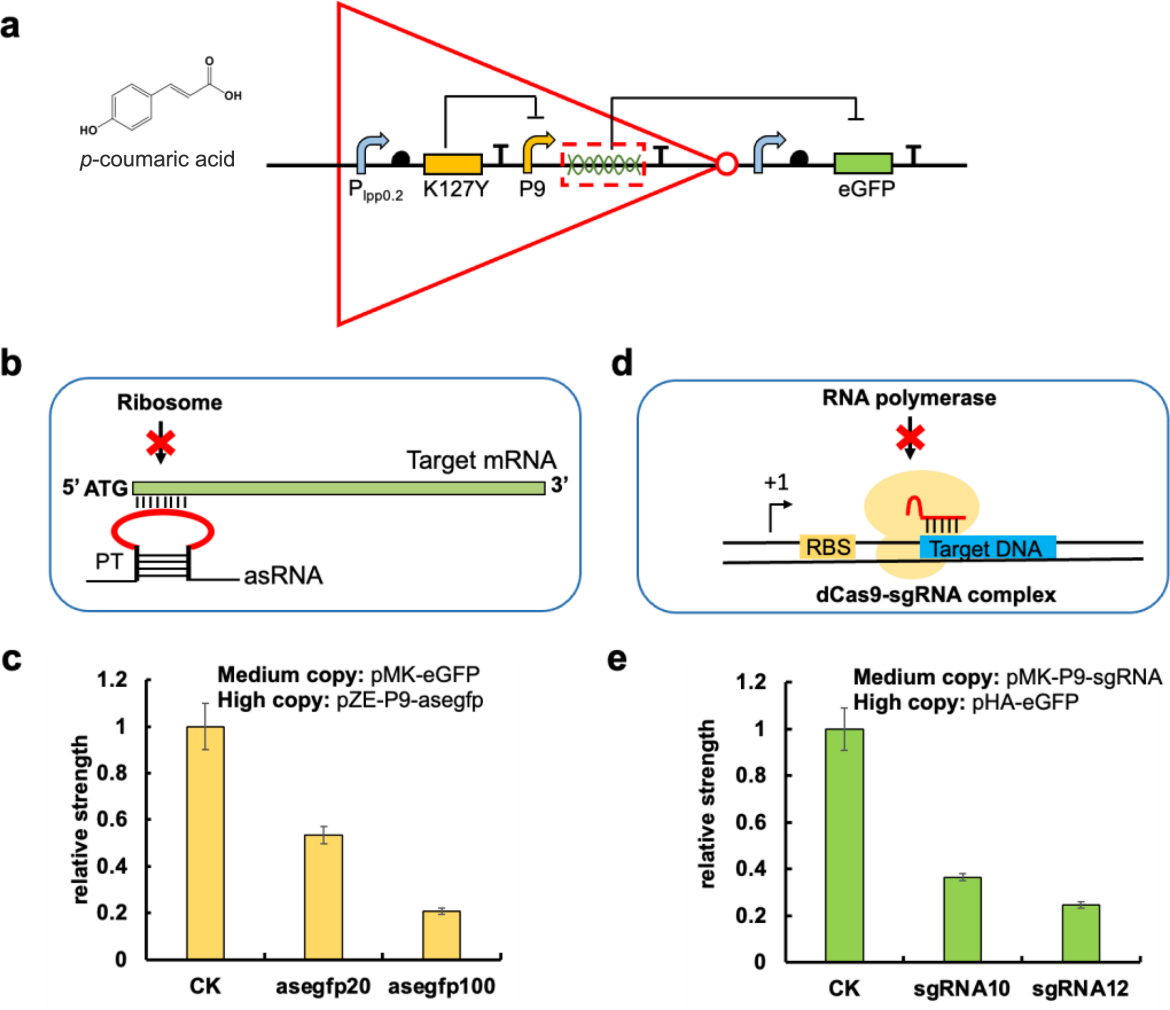
Design of the NOT genetic logic gate. (a) Without the presence of *p*-coumaric acid, the regulatory protein K127Y suppresses the activity of the P9 promoter and asRNA/sgRNA transcription, resulting in normal expression levels of the reporter gene *egfp*. However, the introduction of *p*-coumaric acid nullifies the repression of K127Y on P9, causing an increase in the level of small RNA transcription. These small RNAs interfere with the expression of eGFP, leading to a decrease in its expression levels. (b) The mechanism of asRNA repression. The asRNA with PT structure in both the left and right sides can interact with the complementary sequence of target mRNA and block the access of ribosome, repressing gene translation. (c) The repression efficiency of asRNA in high copy plasmid toward eGFP expressed in medium copy plasmid. CK: without asRNA repression; asegfp20:20 bp antisense RNA was designed to target the eGFP in medium copy plasmids; asegfp100:100 bp antisense RNA was designed to target the eGFP in medium copy plasmids. (d) Mechanism of sgRNA repression. The dCas9-sgRNA complex can recognize the target DNA sequence and block the access of RNA polymerase to repress gene transcription. (e) The repression efficiency of sgRNA in medium copy number plasmid toward eGFP expressed in high copy plasmid. CK: without sgRNA repression; sgRNA10:10 bp antisense RNA was designed to target the eGFP in high copy plasmids; sgRNA12:12 bp antisense RNA was designed to target the eGFP in high copy plasmids. All data are reported as mean ± s.d. from three biologically independent experiments (*n* = 3). Error bars are defined as s.d.

NOT logic gate produces an output to invert its input. Specifically, when the input is a logical one, the NOT gate outputs a logical zero, and when the input is a logical zero, it outputs a logical one. Similarly, in genetic logic gates, a NOT gate can be designed based on the BUF gate, which is a conventional logic gate that activates downstream gene expression when specific inducers are present. To create a NOT genetic logic gate, the logic should be designed so that the addition of inducers turns off gene expression rather than activating it. By implementing an input-triggered promoter to control asRNA or sgRNA, the presence of an input signal will trigger the expression of asRNA or sgRNA, which represses the expression of the target gene (Figure 7a). As the input concentration increases, the repression of gene expression becomes more pronounced, resulting in the formation of a NOT genetic logic gate.

**Figure 8:**
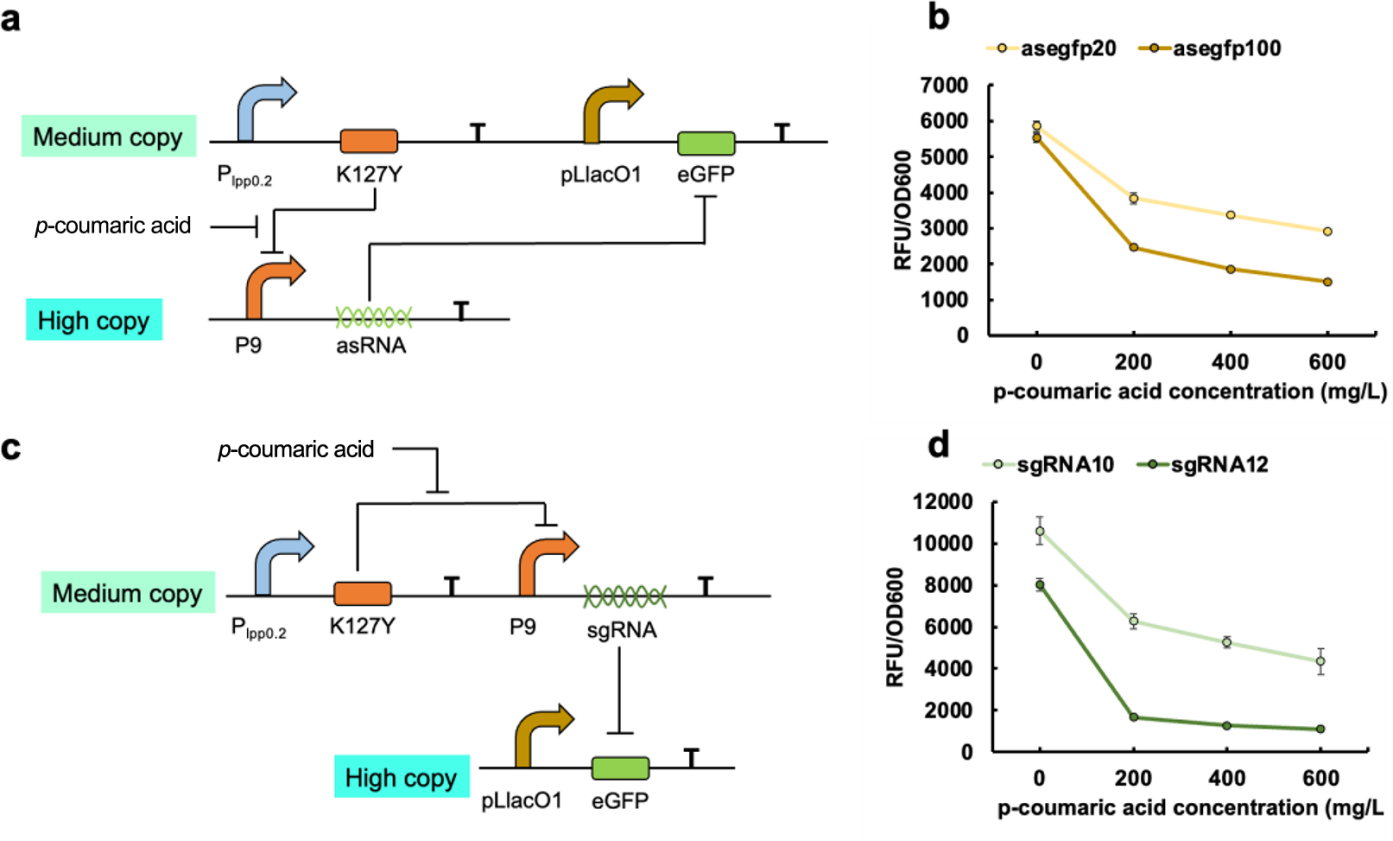
Characterization of the NOT genetic logic gate. (a) The plasmid constitution of the asRNA-based NOT genetic logic gate. (b) The dynamic performance of the asRNA-based NOT genetic logic gate. (c) The plasmid constitution of sgRNA-based NOT genetic logic gate. (d) The dynamic performance of sgRNA-based NOT genetic logic gate. All data are reported as mean ± s.d. from three biologically independent experiments (*n* = 3). Error bars are defined as s.d.

Antisense RNA repression has emerged as a popular approach in metabolic engineering for modulating gene expression. This strategy involves using small complementary RNAs to interfere with target gene expression at the post-transcriptional level. In our previous research, various asRNAs have been designed to target gene repression.^27^ Based on the design principle, we constructed two asRNAs (pZE-P9-asegfp20/100) that target the *egfp* gene in medium copy number plasmid pMK-eGFP-MCS, complementing the 5′ end of the mRNA with varying lengths and repression efficiencies, as illustrated in Figure 7b,c. By integrating the asRNA into *p*-coumaric acid-triggered BUF gate, the K127Y regulator can repress the promoter P9-controlled asRNA transcription, and eGFP can express successfully (Figure 8a). With the increased concentration of *p*-coumaric acid, K127Y repression on P9 promoter was released and asRNA starts transcription to repress the *egfp* gene, showing decreased expression level (Figure 8b). Due to the difference in repression efficiency, the NOT gate with asegfp20 and asegfp100 showed various dynamic repression performance. With 600 mg/L *p*-coumaric acid, the NOT gates with asegfp20 and asegfp100 turned off 50% and 73% of the eGFP expression compared with the group without *p*-coumaric acid. Such results indicated that the incorporation of asRNA into the *p*-coumaric acid system effectively transformed the BUF gate into an NOT gate.

In contrast to asRNA, the repression mediated by sgRNA occurs at the transcriptional level. sgRNA serves as a pivotal engineered component within the Type-II CRISPR (clustered regularly interspaced short palindromic repeat) system, which is naturally found in *Streptococcus pyogenes*. This system has been adapted for synthetic gene repression, functioning by binding to complementary DNA regions and obstructing the interaction of the RNA polymerase with the specific DNA sequence (Figure 7d). To elaborate, we designed two sgRNAs (pMK-P9-sgRNA10/sgRNA12) based on previous research to target the *egfp* gene within a high copy number plasmid pHA-eGFP-MCS, as depicted in Figure 7e. These different lengths of sgRNAs, which exhibited diverse levels of repression efficiencies, were integrated into *p*-coumaric acid-triggered BUF gates, thereby forming NOT gates (Figure 8c). Analogously, the constructed NOT gates demonstrated substantial eGFP expression in the absence of *p*-coumaric acid. However, upon the introduction of *p*-coumaric acid, the NOT gates gradually attenuated eGFP expression in the high copy number plasmid. Compared to the group without inducer, the introduction of 600 mg/L *p*-coumaric acid led to a reduction of eGFP expression by as much as 61% and 87% with sgRNA10 and sgRNA12, respectively (Figure 8d). By utilizing distinct asRNAs and sgRNAs with varying repression efficiencies, the resultant NOT gates exhibited a versatile range of dynamic performances. This enables their integration into various *p*-coumaric acid-derived pathways, thereby facilitating the achievement of different levels of repression efficiencies.

### 3.4 The Construction of Orthogonal Bifunctional Circuits to Control Gene Expression

**Figure 9:**
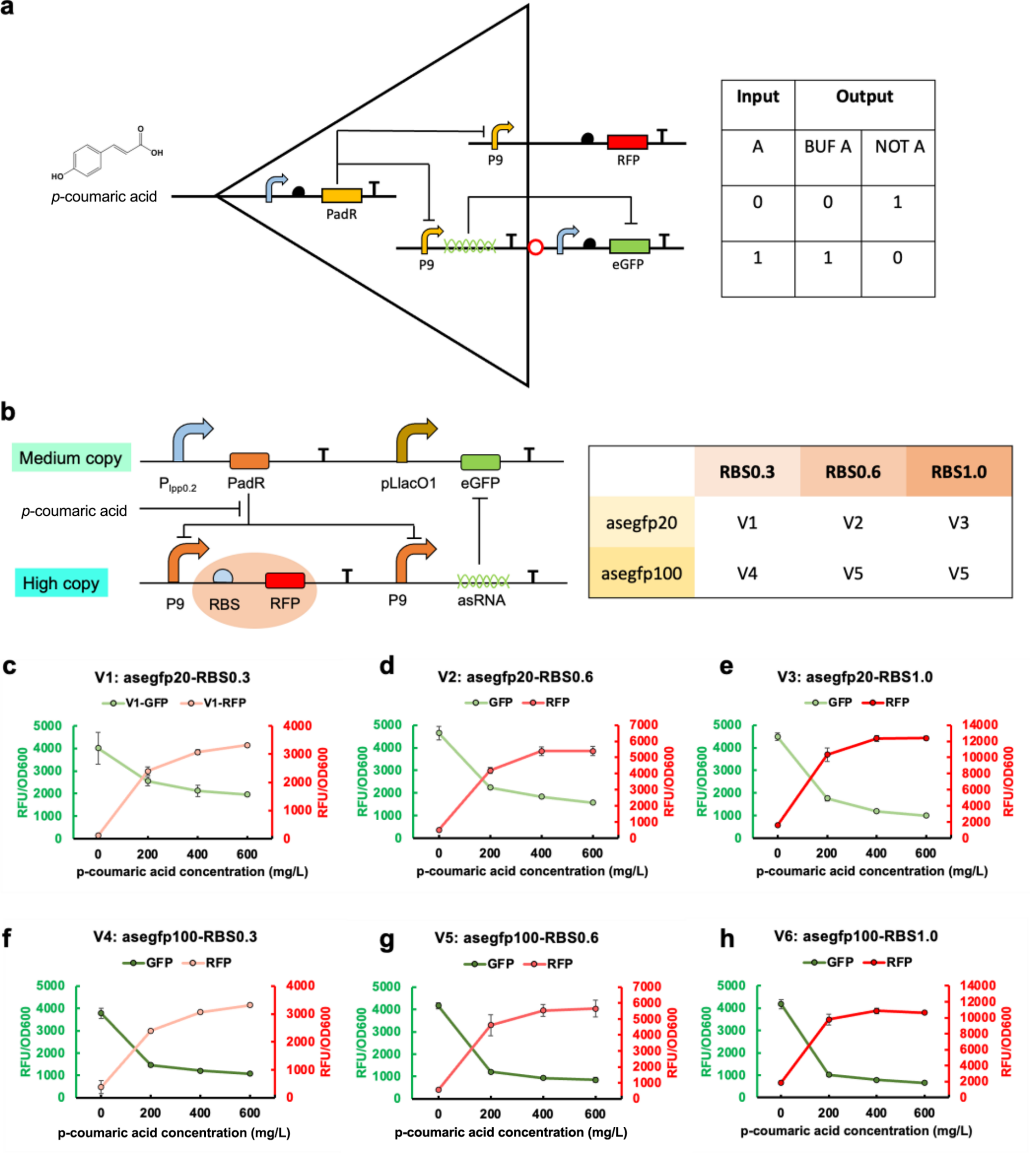
Design and characterization of the asRNA-based bifunctional control. (a) The mechanism of bifunctional control. The activation module utilizes promoter P9 to control the expression of RFP, while the repression module utilizes asRNA/sgRNA controlled by promoter P9 to repress eGFP expression. In the absence of the inducer *p*-coumaric acid, the regulator K127Y represses promoter P9, resulting in the repression of both RFP and asRNA/sgRNA expression that leads to eGFP expression. Upon the addition of *p*-coumaric acid, K127Y repression is released, resulting in the activation of both RFP and asRNA/sgRNA expression. At the same time, the repression of eGFP is triggered by the small RNA. (b) The plasmid constitution of the asRNA-based bifunctional control. (c) V1: the combination of an asegfp20-based NOT gate and a BUF gate with RBS0.3 before the *rfp* gene. (d) V2: the combination of an asegfp20-based NOT gate and a BUF gate with RBS0.6 before the *rfp* gene. (e) V3: the combination of an asegfp20-based NOT gate and a BUF gate with RBS1.0 before the *rfp* gene. (f) V4: the combination of an asegfp100-based NOT gate and a BUF gate with RBS0.3 before the *rfp* gene. (g) V5: the combination of an asegfp100-based NOT gate and a BUF gate with RBS0.6 before the *rfp* gene. (h) V6: the combination of an asegfp100-based NOT gate and a BUF gate with RBS1.0 before the *rfp* gene. All data are reported as mean ± s.d. from three biologically independent experiments (*n* = 3). Error bars are defined as s.d.

In natural biological systems, gene activation and repression controlled by the same regulators sometimes occur simultaneously. To construct more complex genetic circuits and achieve bifunctional control of gene expression, it is essential to investigate the orthogonality of the genetic logic gates. In order to explore the feasibility of bifunctional control, we used a fluorescent reporter gene *rfp*, regulated by the *p*-coumaric acid-induced promoter P9, to assess gene activation in the BUF gate. By combination of the BUF gate with a NOT gate where eGFP was used as the reporter, regulator K127Y can repress the P9 promoters in both the BUF and NOT gates. Theoretically, the absence of *p*-coumaric acid turns off the BUF and NOT gate, resulting in the repression of RFP and the expression of eGFP. Conversely, when *p*-coumaric acid is present, the K127Y repression toward the two P9 promoters is alleviated, leading to the activation of RFP and the repression of eGFP. Thus, this circuit can produce two distinct outputs from a single input (Figure 9a).

As a proof-of-concept, we integrated RNA-based NOT gates with three different BUF gates in one cell (Figure 9b). This yielded six versions of bifunctional control circuits. As expected, when *p*-coumaric acid was absent, the BUF gates were repressed, leading to no RFP expression. Simultaneously, the NOT gates can express eGFP successfully. With increased *p*-coumaric acid concentrations, the BUF and NOT gates were activated, leading to RFP expression and suppression of eGFP (Figure 9c–h). These results demonstrated the successful construction of bifunctional genetic circuits which are controlled by one inducer. Furthermore, no matter which BUF gates were combined with the asegfp20-based NOT gate, it can function normally without interference (Figure 9c,d,e). The asegfp100-based NOT gate exhibited consistent performance when combined with different versions of BUF gates (Figure 9f,g,h). Similarly, regardless of whichever NOT gates were integrated, the BUF gates can operate independently, showing consistent dynamic performance (Figure 9c,f, Figure 9d,g, Figure 9e,h). Taken together, the amalgamation of BUF gates with asRNA-based NOT gates resulted in bifunctional control genetic circuits, which exhibited gradually changing activation and repression patterns upon the introduction of different concentrations of *p*-coumaric acid. Moreover, our results showcased that the BUF and NOT gates can function normally, and the dynamic performance of each gate has not been affected by each other. This ability to achieve bifunctional control by integrating BUF and NOT gates holds significant promise for advancing genetic circuit development in *p*-coumaric acid-derived pathways to regulate gene activation and repression simultaneously.

## Conclusion

Standard genetic logic gates are vital for creating robust genetic circuits in metabolic engineering and synthetic biology, which offer customizable cell behavior and pathway regulation. This paper explored the parameters impacting *p*-coumaric acid-triggered BUF gates and concluded that regulating the output gene is an effective strategy for designing versatile BUF gates with adjustable dynamic performance. Additionally, we designed AND genetic logic gates by combining the binding sequences of K127Y and tetR/lacI into one promoter. When both K127Y and tetR, or both K127Y and lacI, bind to the hybrid promoters P9-tetR or P9-lacI, respectively, the promoters were repressed, and only the simultaneous presence of *p*-coumaric acid and tetracycline, or *p*-coumaric acid and IPTG, can activate the promoter and initiate gene expression. Furthermore, we devised *p*-coumaric acid-triggered NOT gates by combining characterized asRNAs and sgRNAs with BUF gates. This approach enabled the opening of NOT gates for gene repression upon *p*-coumaric acid addition. To construct bifunctional genetic circuits and assess orthogonality, we combined the designed BUF and NOT gates within a single cell. Remarkably, this demonstrated that K127Y can repress both gates and that *p*-coumaric acid can trigger the closing and opening of related logic gates independently. These efforts introduce new inspirations for the progression of genetic logic gates, expanding their potential applications in metabolic engineering and synthetic biology.
